# Classification of 41 Hand and Wrist Movements via Surface Electromyogram Using Deep Neural Network

**DOI:** 10.3389/fbioe.2021.548357

**Published:** 2021-06-09

**Authors:** Panyawut Sri-iesaranusorn, Attawit Chaiyaroj, Chatchai Buekban, Songphon Dumnin, Ronachai Pongthornseri, Chusak Thanawattano, Decho Surangsrirat

**Affiliations:** ^1^Mathematical Informatics, Information Science, Nara Institute of Science and Technology, Nara, Japan; ^2^Assistive Technology and Medical Devices Research Center, National Science and Technology Development Agency, Pathum Thani, Thailand

**Keywords:** surface electromyogram, hand movement classification, deep neural network, prosthetic hand, Ninapro database

## Abstract

Surface electromyography (sEMG) is a non-invasive and straightforward way to allow the user to actively control the prosthesis. However, results reported by previous studies on using sEMG for hand and wrist movement classification vary by a large margin, due to several factors including but not limited to the number of classes and the acquisition protocol. The objective of this paper is to investigate the deep neural network approach on the classification of 41 hand and wrist movements based on the sEMG signal. The proposed models were trained and evaluated using the publicly available database from the Ninapro project, one of the largest public sEMG databases for advanced hand myoelectric prosthetics. Two datasets, DB5 with a low-cost 16 channels and 200 Hz sampling rate setup and DB7 with 12 channels and 2 kHz sampling rate setup, were used for this study. Our approach achieved an overall accuracy of 93.87 ± 1.49 and 91.69 ± 4.68% with a balanced accuracy of 84.00 ± 3.40 and 84.66 ± 4.78% for DB5 and DB7, respectively. We also observed a performance gain when considering only a subset of the movements, namely the six main hand movements based on six prehensile patterns from the Southampton Hand Assessment Procedure (SHAP), a clinically validated hand functional assessment protocol. Classification on only the SHAP movements in DB5 attained an overall accuracy of 98.82 ± 0.58% with a balanced accuracy of 94.48 ± 2.55%. With the same set of movements, our model also achieved an overall accuracy of 99.00% with a balanced accuracy of 91.27% on data from one of the amputee participants in DB7. These results suggest that with more data on the amputee subjects, our proposal could be a promising approach for controlling versatile prosthetic hands with a wide range of predefined hand and wrist movements.

## 1. Introduction

Recent advancements in sensor technology, mechatronics, signal processing techniques, and edge computing hardware equipped with GPU make dexterous prosthetic hands with non-invasive sensors and control capabilities of machine learning possible. However, real-world applications of these prostheses and amputees' receptions of them are still limited. Some of the main reasons include control difficulties, insufficient capabilities and dexterity levels, and the cost of the prosthesis. Moreover, frequent misclassifications of intended actions could lead to frustration and prostheses abandonment (Biddiss and Chau, 2007; Ahmadizadeh et al., 2017). Therefore, achieving a high level of reliability and robustness of human-machine interfaces is important for user experience and their acceptance of the prosthetic hand.

**Figure 1:**
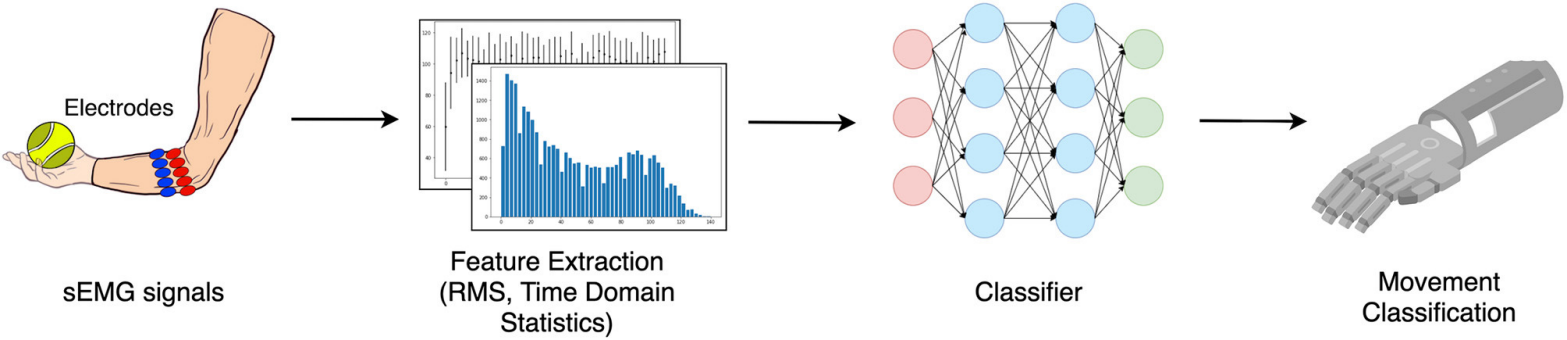
Overview of hand movement classification using sEMG.

Over the last few years, multiple non-invasive control methods of prosthetic hands have been introduced and investigated; for example, surface electromyography (sEMG) (Fougner et al., 2012; Farina et al., 2014; Krasoulis et al., 2017; Pizzolato et al., 2017; Ameri et al., 2018; Li et al., 2018; Leone et al., 2019; Olsson et al., 2019; Junior et al., 2020), electroneurography (ENG) (Cloutier and Yang, 2013; Paul et al., 2018), mechanomyography (MMG) (Xiloyannis et al., 2015; Wilson and Vaidyanathan, 2017), and force myography (FMG) (Rasouli et al., 2015; Sadeghi and Menon, 2018; Ahmadizadeh et al., 2019). In particular, sEMG is a non-invasive technique for measuring the electrical activity of groups of muscles on the skin surface, which makes it a simple and straightforward way to allow the user to actively control the prosthesis. The overview of hand movement classification for the prosthetic hand using sEMG is shown in Figure 1. The muscle signals are collected as an input for the movement classification. The process typically involves feature extraction and classification process by the selected classifier.

Ameri et al. (2018) performed an sEMG classification of wrist movements based on the raw signal without any feature extraction using Support Vector Machine (SVM) and Convolutional Neural Network (CNN). The data was collected from 17 healthy individuals using eight pairs of bipolar surface electrodes with 1.2 kHz sampling rate equally spaced around the dominant forearm proximal to the elbow. A total of eight wrist movements were investigated. The classification results for the CNN and SVM were 91.61 ± 0.39 and 90.63 ± 0.31%, respectively. Li et al. (2018) investigated the use of sEMG for the classification of grasping force of a three-finger pinch movement for prosthetic control. The grasping force was separated into eight levels. A total of 15 healthy participants were recruited for the experiment. The signal was collected using a Thalmic Myo armband with 8 sEMG input channels and a 200 Hz sampling rate. Principal Component Analysis (PCA) was implemented to reduce the dimension of the extracted features to shorten the computation time. The force classification accuracy was over 95% with between-subject variations ranged from 3.58 to 1.25%. Leone et al. (2019) presented classification results for both hand or wrist gestures and forces. The algorithm was evaluated on 31 healthy participants for seven movements using commercial sEMG sensors, Ottobock 13E2000, providing six channels of input and a sampling rate of 1 kHz. The best average accuracy of 98.78% was achieved with non-linear logistic regression (NLR) algorithm. Olsson et al. (2019) experimented with a high-density sEMG (HD-sEMG) for the classification of 16 hand movements using CNN. HD-sEMG signal was collected using two of the eight-by-eight electrode arrays coated with conductive gel, for a total of 128 input channels. Fourteen healthy adults participated in this study. The input size for the CNN model was 16 × 8 × 24, 24 HD-sEMG samples. The classification accuracy was 78.13 ± 6.80% with individual subject accuracy ranged from 62 to 85%. Junior et al. (2020) investigated multiple classification techniques for six hand gestures acquired from 13 participants using eight channels sEMG armband with a sampling rate of 2 kHz. Their best result with the average accuracy of 94% was obtained from 40 features with the large margin nearest neighbor (LMNN) technique. Côté-Allard et al. (2020) presented an analysis of the features learned using deep learning for the classification of 11 hand gestures using sEMG. They concluded that handcrafted features and learned features could discriminate between the gestures but do not encode the same information. The learned features tend to ignore the most activated channel while the handcrafted features were designed to capture the amplitude information. The authors also presented an Adaptive Domain Adversarial Neural Network (ADANN) designed to study learned features that generalize well across participants. The dataset used in this study included 22 healthy participants performing ten hand and wrist gestures using the 3DC armband with ten channels at a 1 kHz sampling rate. The average accuracy was 84.43 ± 0.05% for the 10 movements. Krasoulis et al. (2017) and Pizzolato et al. (2017) performed an sEMG classification of over 40 hand and wrist movements. Krasoulis et al. (2017) reported average accuracy scores for 20 participants in the able-bodied group at 63%. For two participants in the amputee group, the average accuracy scores were 60%. Both experiments used linear discriminant analysis (LDA) classifier for movement intent decoding. Pizzolato et al. (2017) reported the best results with an accuracy of 74.01 ± 7.59% for the 41 movements in the group of 40 able-bodied participants using random forest classifier on hand-crafted features.

Recent research on the sEMG classification using a deep learning approach tends to gravitate toward using CNN to automatically learn the features from a raw signal. However, training a deep neural network generally requires a large amount of training data for it to converge and discover meaningful features, especially for CNN. Moreover, CNN has a relatively high memory cost and processing time, which may pose challenges when running on embedded systems with limited resources. For our experiment, we were concerned about the limited amount of training data for the classification of 41 movements. Also, we would like to investigate the feasibility of adopting an accurate deep learning approach that would be able to run on affordable hardware. Therefore, we chose to extract hand-crafted statistical features and feed them to our deep neural network (DNN) model for the classification.

Even though the classification of hand and wrist movements using sEMG has been investigated and reported by multiple research teams, the classification results described in the literature can vary by a large margin, ranging from 60 to 98% accuracy. The results depend on several parameters, such as the number of classes, the number of samples, the acquisition protocol and setup, and the evaluation metrics. Hence, for qualitative comparison, the experimental results should consider only studies targeting a similar number of classes, where the chance levels are comparable.

The objective of this study is to investigate the DNN approach for the classification of the hand and wrist movements based on the sEMG signal. The experiments considered 41 movements of Hand, wrist, grasping, and functional hand movements. Feature extraction techniques on the sEMG signal were explored and selected for the best balance between classification performance and computational complexity. The result of the proposed deep neural network classifier was validated on the publicly accessible datasets from the Ninapro database, one of the largest public sEMG databases for advanced hand myoelectric prosthetics. The Ninapro project is an ongoing work that aims to create an accurate and comprehensive reference for scientific research on the relationship between sEMG, hand or arm kinematics, and dynamics, and clinical parameters, with the final goal of creating non-invasive, naturally controlled robotic hand prostheses for transradial amputees (Atzori et al., 2014). The data used in Krasoulis et al. (2017) and Pizzolato et al. (2017) experiments were also collected according to the protocol described in the Ninapro project and the data were included in the Ninapro database. At the time of writing, the project consists of eight datasets with a predefined set of up to 53 movements. A summary of the database is shown in Table 1. In this study, Ninapro DB5 and DB7 were chosen since they are the two newest datasets with comparable data collection protocols for 41 movements. The acquisition setup for DB5 is based on two Thalmic Myo armbands with 16 sEMG input channels and a 200 Hz sampling rate, which cost a few hundred dollars compared to several thousand dollars for other setups. The acquisition setup for DB7 is based on 12 sEMG input channels of Delsys Trigno electrodes with a 2 kHz sampling rate. The performance of our proposed technique was compared with the best performance from the previous study on the dataset.

**Table 1:** Overview of the datasets in the Ninapro project.

	**DB1**	**DB2**	**DB3**	**DB4**	**DB5**	**DB6**	**DB7**	**DB8**
Intact participants	27	40	–	10	10	10	20	10
Amputees	–	–	11	–	–	–	2	2
Repetitions	10	6	6	6	6	70	6	12
Movements	53	50	50	53	53	7	41	9
Sensors	Otto	Delsys	Delsys	Cometa	Myo	Delsys	Delsys	Delsys
Sampling rate	100 Hz	2 kHz	2 kHz	2 kHz	200 Hz	2 kHz	2 kHz	2 kHz

The contributions of this study are as follows: (1) performance improvement for the classification of sEMG for 41 hand and wrist movements; (2) performance comparison between the sEMG setups of low cost and low sampling rate sensors—double Myo armbands with 16 input channels, and high cost and high sampling rate sensors—12 input channels of the Delsys Trigno electrodes; (3) performance comparison of multiple decision window sizes ranging between 100 and 1,000 ms. All of the experiments were validated using the publicly available database.

## 2. Materials and Methods

### 2.1. Database and Acquisition Setup

**Figure 2:**
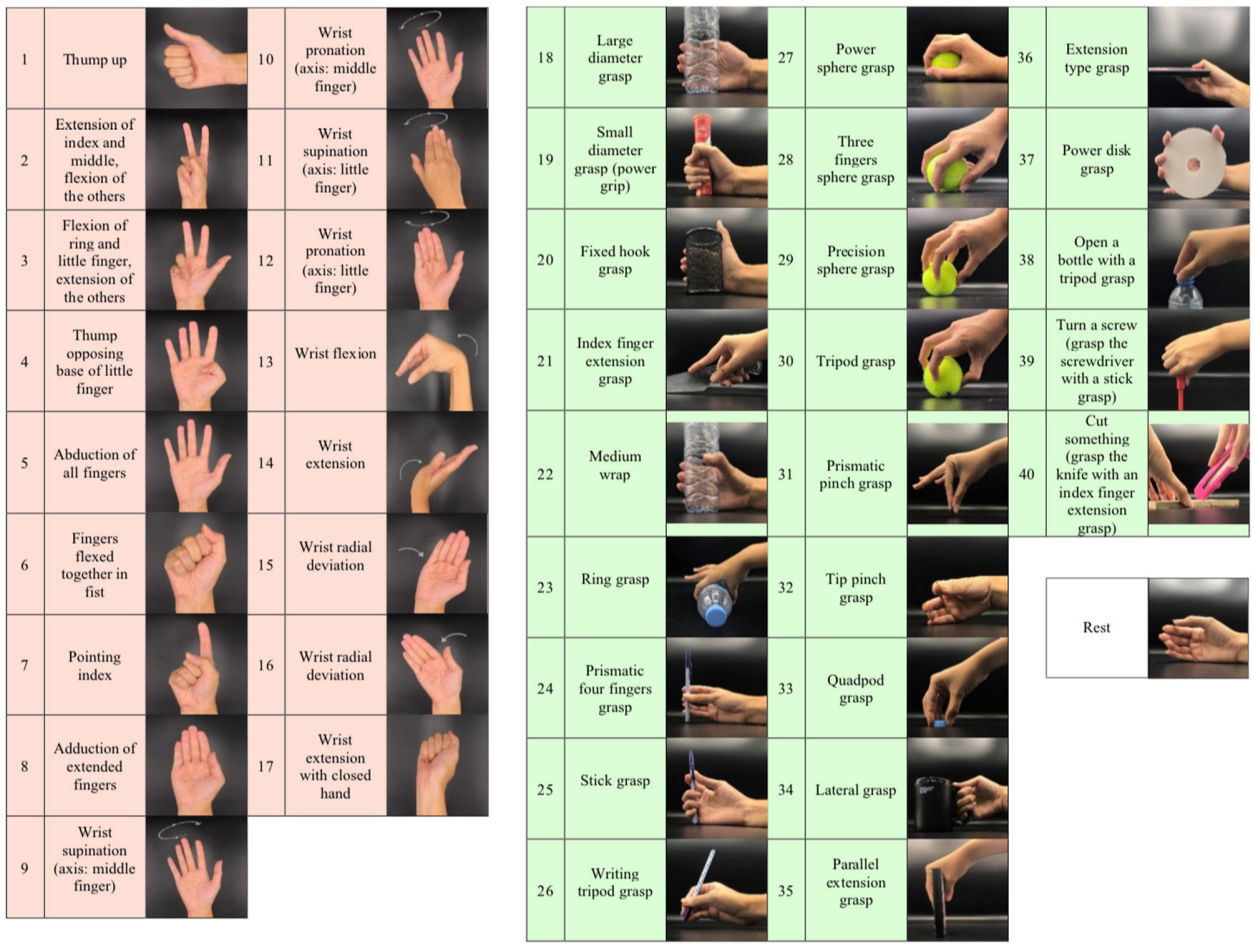
Illustration of the 41 movements in this study according to the grouping from the Ninapro project: isometric and isotonic hand configurations and basic wrist movements (17 exercises), grasping and functional movements (23 exercises), and rest position.

The database of the Ninapro project was used in this study. Ninapro DB5 and DB7, two of their newest datasets acquired using the same data acquisition protocols, were selected for comparison. For the data acquisition protocol, participants were instructed to repeat several hand movements by following videos shown on a laptop screen. The recording of each movement took 5 s, with 3 s of rest to avoid errors from muscular fatigue. For every hand movement recording, participants performed six repetitions, to account for slight variations of the exact hand muscle movements within the same movement class. DB5 has a total of 53 movements while DB7 has 41 movements. The same movements that were collected in both datasets are from two movement groups: isometric and isotonic hand configurations and basic wrist movements (17 exercises), and grasping and functional movements (23 exercises), as illustrated in Figure 2. According to the Ninapro project, all of the movements were selected from the hand taxonomy as well as from hand robotics literature. Following previous studies on the Ninapro database, we used repetitions 1, 3, 4, and 6 as training data, while repetitions 2 and 5 were used for evaluation (Atzori et al., 2014; Atzori and Müller, 2015; Pizzolato et al., 2017).

For DB5, the low cost and low sampling rate dataset, the sEMG was recorded with two Thalmic Myo armbands. Each Myo armband has eight sEMG electrodes with a sampling rate of 200 Hz. The upper armband is placed closer to the elbow with the first electrode on the radio humeral joint. The lower armband is tilted by 22.5° and placed next to the upper one to fill in the gaps between its electrodes. This setting provides an extended uniform muscle mapping at the most affordable cost. The participants in this dataset are 10 intact participants, eight males and two females, with an average age of 28.00±3.97 years. On the other hand, the high cost and high sampling rate DB7 dataset used 12 Delsys Trigno electrodes for sEMG recording with a sampling rate of 2 kHz. Eight sensors were placed around three centimeters below the elbow and equally spaced around the forearm. Two sensors were placed for the extrinsic hand muscles; Extensor Digitorum Communis (EDC) and Flexor Digitorum Superficialis (FDS). The last two sensors were placed on the biceps and triceps brachii muscles. The dataset contains 20 intact participants and 2 amputee participants, with an average age of 27.73±6.53 years. The first amputee participant was a transradial 28 years old male with 6 years of right limb loss due to a car accident. The second amputee participant was a transradial 54 years old male with 18 years of right limb loss due to epithelioid sarcoma cancer.

**Figure 3:**
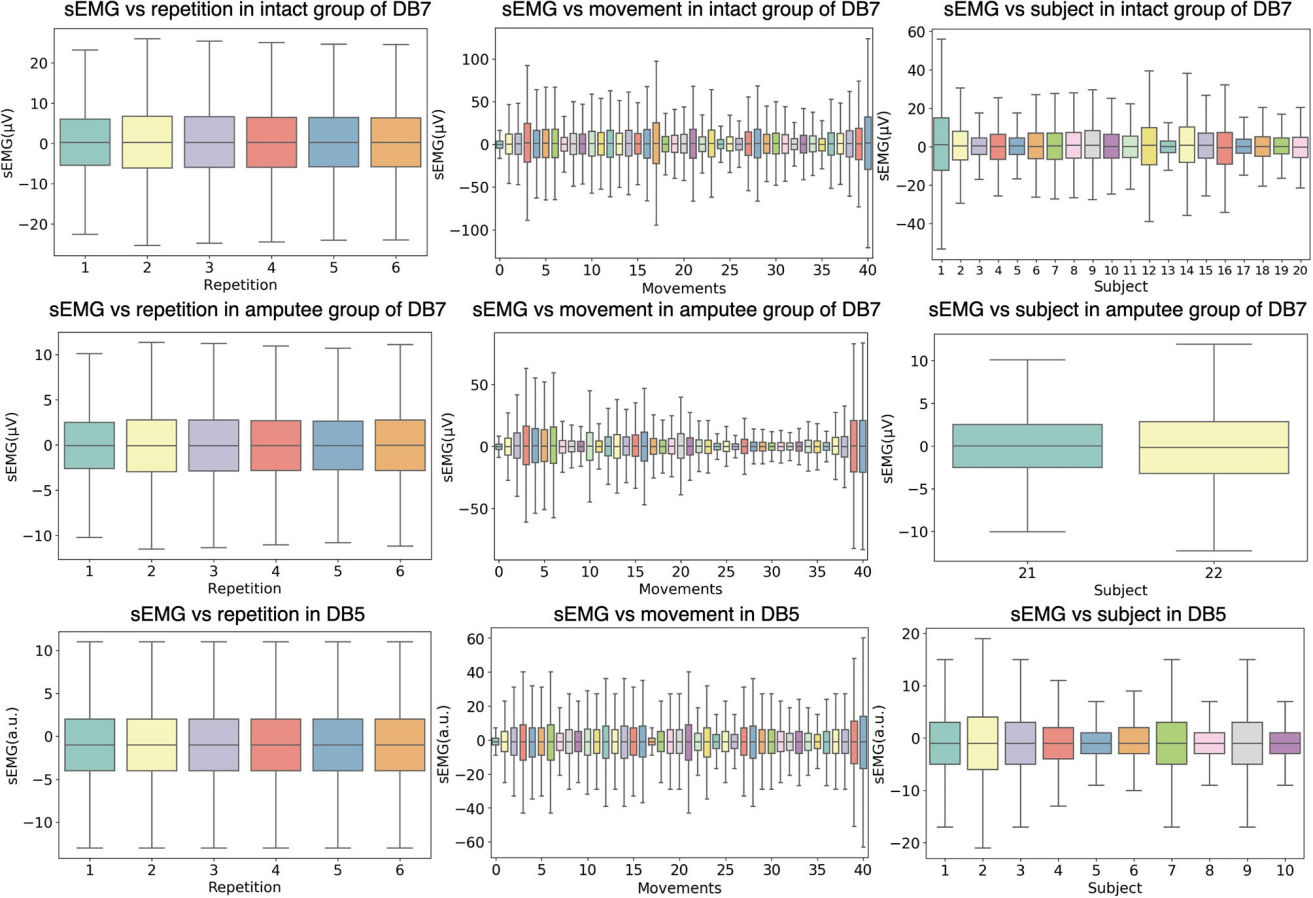
Distributions of the sEMG signal amplitudes grouped by different experiment conditions. The first two rows show data from the intact and amputee groups from DB7, while the third row shows DB5. The first column groups the amplitudes from all movements and subjects by repetition; the second column from all repetitions and subjects by movement; the third column from all repetitions and movements by subject. The horizontal lines in the middle of each box mark the median; the edges denote the first and third quartiles; the whiskers cover approximately 2.7 times the standard deviation.

Figure 3 shows the relationship between the amplitude of the sEMG signal and different experimental conditions, namely movement repetition, movement class, and subject. The data from the first channel of each dataset is illustrated and the outliers are omitted for readability purposes. Even though the subjects performed each repetition under the same environment, the signals may differ between repetitions due to physiological factors such as muscular characteristics, skin impedance, sweat, muscular tone, or fatigue. To statistically validate the differences in each repetition, we conducted a one-way analysis of variance (ANOVA) statistical test on the concatenation of all signal channels in each repetition. Our analysis showed that there are no significant differences between different movement repetitions (*P* > 0.1) for all intact and amputee subjects of DB7 and all subjects in DB5.

### 2.2. Data Preprocessing and Feature Extraction

To process real-time sEMG data, the raw signals were sectioned using a sliding window. To introduce time variation as well as add more samples, the stride between each window was set to be smaller than the window size, resulting in some overlap between consecutive samples. We extracted from each window the following features: the root mean square (RMS), and time-domain statistics as described by Hudgins et al. (1993); mean absolute value, mean absolute value slope, zero crossings, slope sign changes, and waveform length. Each feature was standardized into a normal distribution to make sure no feature is favored unequally over the others due to scale or range.

Out of all the time domain features, zero crossings and slope sign changes were noted to require a noise threshold. Due to being features based on counting occurrences of, for example, the values crossing zero, one must exclude any occurrences caused by low-valued noise. We restate these features more formally as follows. Given a window of data *x*_*a*...*b*_, the zero crossing function *z*(*x*_*k*_) is equal to 1 when:

(1) $$\begin{array}{r} \left[\left(x_{k}<0\text{ and }x_{k+1}>0\right)\text{ or }\left(x_{k}>0\text{ and }x_{k+1}<0\right)\right]\\ \text{ and }\left(|x_{k}-x_{k+1}|\geq T\right) \end{array}$$

where *T* is the noise threshold, and 0 otherwise. The zero crossing feature *zc*(*x*_*a*...*b*_) itself is an accumulation, or a summation of said function:

(2) $$\begin{array}{l} zc\left(x_{a...b}\right)=\overset{b}{\underset{k=a}{\sum }}z\left(x_{k}\right) \end{array}$$

The slope sign change feature is defined similarly. The condition for the slope sign change function *s*(*x*_*k*_) being equal to 1 is:

(3) $$\begin{array}{r} \left[\left(x_{k}>x_{k-1}\text{ and }x_{k}>x_{k+1}\right)\text{ or }\left(x_{k}<x_{k-1}\text{ and }x_{k}<x_{k+1}\right)\right]\\ \text{ and }\left(|x_{k}-x_{k+1}|\geq T\text{ or }|x_{k}-x_{k-1}|\geq T\right) \end{array}$$

and the slope sign change feature *ssc*(*x*_*a*...*b*_):

(4) $$\begin{array}{l} ssc\left(x_{a...b}\right)=\overset{b}{\underset{k=a}{\sum }}s\left(x_{k}\right) \end{array}$$

**Figure 4:**
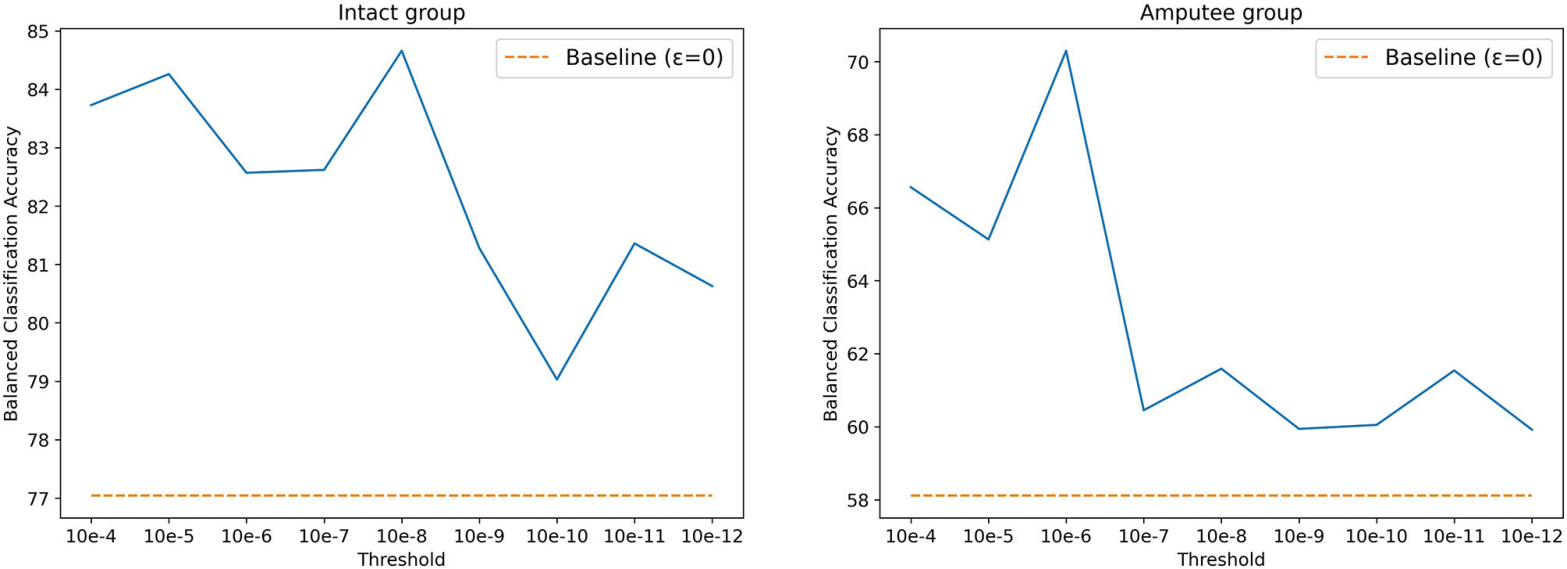
Balanced classification accuracy at different thresholds for the intact and amputee groups from DB7.

The threshold *T* was set to 0.01*V* for DB5, as described in the original paper. Using the same value for DB7, however, yielded poor classification results. According to Kamavuako et al. (2016), the optimal threshold is usually data- and subject-driven, and does not generalize well. However, selecting a threshold based on the dataset's signal to noise ratio can still significantly increase the classification accuracy. Following this statement, we searched for the optimal threshold for DB7 by performing a grid search, setting thresholds between 10^−4^ and 10^−12^, increasing by a factor of 10. As shown in Figure 4, 10^−8^ and 10^−6^ are the best threshold parameters for intact and amputee groups, respectively. Similar to prior works, the results with threshold yield better accuracy. Thus, we selected 10^−8^ and 10^−6^ as thresholds for intact and amputee groups from DB7, respectively.

### 2.3. Deep Neural Network Classifier

**Figure 5:**
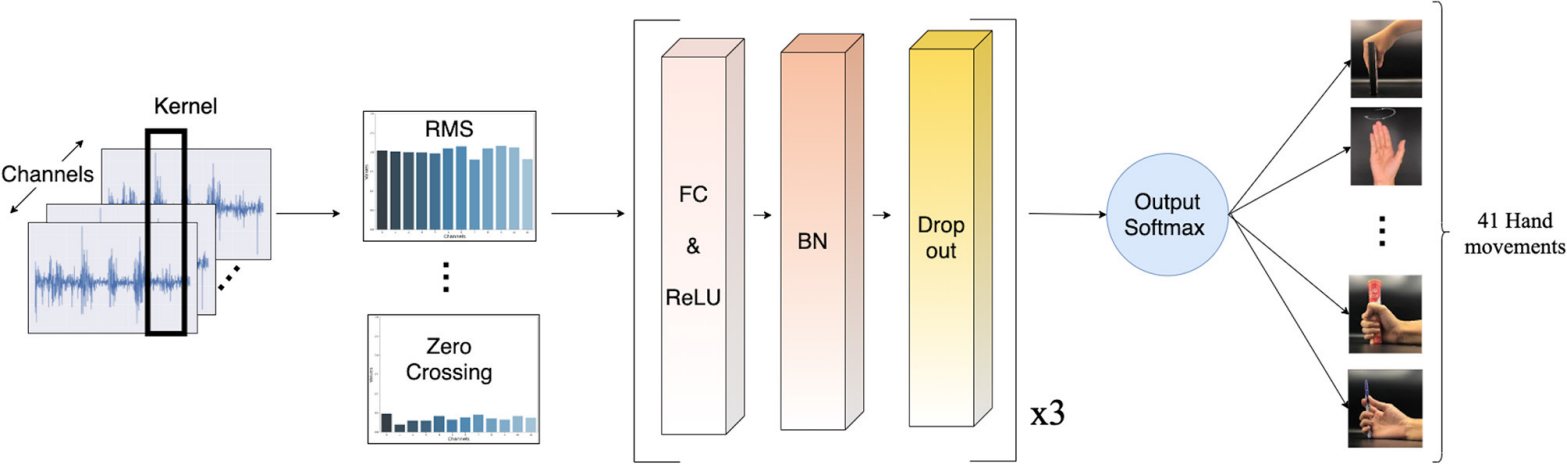
Schematic of the proposed deep neural network model. The sEMG input is segmented by a sliding window. Then, the features are extracted and normalized before passing into the classifier. Lastly, a softmax activation function turns the classifier's output into a probability-like vector for the classification of 41 movements.

A deep neural network (DNN) has been chosen for dealing with real-world signal processing tasks, due to its outstanding performance compared to other machine learning algorithms (Park and Lee, 2016; Chen et al., 2017; Orjuela-Cañón et al., 2017; Tsinganos et al., 2018; Chaiyaroj et al., 2019). Motivated by this fact and considering our aim for a real-time system, we implemented a simple feed-forward neural network model. The model consists of three hidden layers, which are fully connected layers with 512, 256, and 256 neurons, respectively. All layers were initially assigned random weights using the He uniform initialization scheme (He et al., 2015). Each layer is followed by the rectified linear unit (ReLU) activation function, to address the vanishing gradient problem (Nair and Hinton, 2010; Dahl et al., 2013; Nwankpa et al., 2018). For regularization, we applied batch normalization to increase the numerical stability of the neural network, and 20% dropout to prevent over-fitting by forcing the model not to rely on the same patterns all the time (Srivastava et al., 2014; Ioffe and Szegedy, 2015). The output layer uses the softmax activation function to simulate a probability vector, as our task is a multi-class classification. The model was optimized with the Adam optimizer, with a learning rate of 0.005 and decay of 0.00001. Our proposed model is illustrated in Figure 5.

In Equation (5), given a vector of preprocessed signal input *x* and trained weights θ, [*f*_θ_(*x*)]_*k*_ is an output value obtained from passing the input through our DNN model. This output value can be described as a score it assigns to whether the input belongs to each class *k*. To derive a probability vector from the output values of all classes, we added the softmax function in our last output layer. After that, according to Equation (6), the class with the highest probability is selected as the final output.

(5) $$\begin{array}{l} P\left(class=k|x,\theta \right)=\frac{\exp\left({\left[f_{\theta }\left(x\right)\right]}_{k}\right)}{\sum_{i=1}^{n}\exp\left({\left[f_{\theta }\left(x\right)\right]}_{i}\right)} \end{array}$$

(6) $$\begin{array}{l} y=\underset{i}{\mathrm{argmax}}P\left(class=i|x,\theta \right) \end{array}$$

(7) $$\begin{array}{l} L_{CE}=-\overset{N}{\underset{i}{\sum }}t_{i}log\left(s_{i}\right) \end{array}$$

where *t*_*i*_ is either 1 in case the sample's ground truth label is *i*, or otherwise 0, while *s*_*i*_ is the probability score of the sample about which class the model predicts it to belong to.

### 2.4. Evaluation Metrics

According to the data acquisition protocol from Ninapro, data for the rest class was collected after every hand movement exercise to avoid errors from muscular fatigue due to that particular exercise. Therefore, with approximately half of the samples belonging to the rest class, robust and efficient evaluation metrics are necessary to deal with the imbalanced data. Otherwise, the result will not reflect the real performance of the model; the model might perform well just because it outputs only the majority class. For binary classification tasks, a distinction is often made between **overall** accuracy, and **balanced** accuracy. Overall accuracy, often simply referred to as accuracy, is one of the most commonly used metrics, reflecting the number of all correctly identified samples out of all samples. However, this metric does not distinguish samples between classes; thus, it may not show the true performance of the classifier when a class imbalance is present in the data. On the other hand, balanced accuracy, also known as the Balanced Classification Rate (BCR) (Hardison et al., 2008; Brodersen et al., 2010; Tharwat, 2018), can mitigate the imbalance's effect by normalizing the accuracy of each class with the number of samples of the class. In the case of multi-class classification, taking the average of recall values can be generalized as the **macro recall**:

(8) $$\begin{array}{l} macro\text{\_}recall=\frac{1}{N}\overset{N}{\underset{k}{\sum }}recall_{k} \end{array}$$

where *recall*_*k*_ is the percentage of total relevant results correctly classified by our algorithm for class *k*, and *N* is the number of classes. While balanced accuracy is not defined for multi-class classification, we may refer to macro recall as such due to the similarity and to be more in line with other studies. Since macro recall can represent a classifier's performance on each class equally, we have included it along with accuracy as metrics by which the classifiers will be evaluated. To facilitate any comparisons in further studies, we have also included other macro-averaged metrics: macro precision, and macro F1 score. Macro precision is defined as:

(9) $$\begin{array}{l} macro\text{\_}precision=\frac{1}{N}\overset{N}{\underset{k}{\sum }}precision_{k} \end{array}$$

where *precision*_*k*_ is the percentage of predictions that come from class *k*. Macro F1 score is simply the harmonic mean of precision and macro recall:

(10) $$\begin{array}{l} macro\text{\_}f1=2\cdot \frac{macro\text{\_}precision\cdot macro\text{\_}recall}{macro\text{\_}precision+macro\text{\_}recall} \end{array}$$

### 2.5. Usage Simulation

Since our experiments used public datasets from the Ninapro project, we currently do not have a similar acquisition setup for online testing. Therefore, we examine how our system would perform in a real use case using the full sequence from each repetition to simulate usage. Adhering to Ninapro's sEMG data acquisition procedure, we tested our model on the entire lengths of the test repetitions from each intact subject. This procedure illustrates whether the model's predictions will translate into smooth and uninterrupted hand movements. Ideally, the predictions on this dataset should be a period of rest, followed by a continuous sequence of one of the specified hand movements. Any wrong classification in the middle of the sequence indicates that our system will execute an incorrect movement and interrupt the user's intended movement.

## 3. Results

### 3.1. Classification Performance—Intact Group

**Figure 6:**
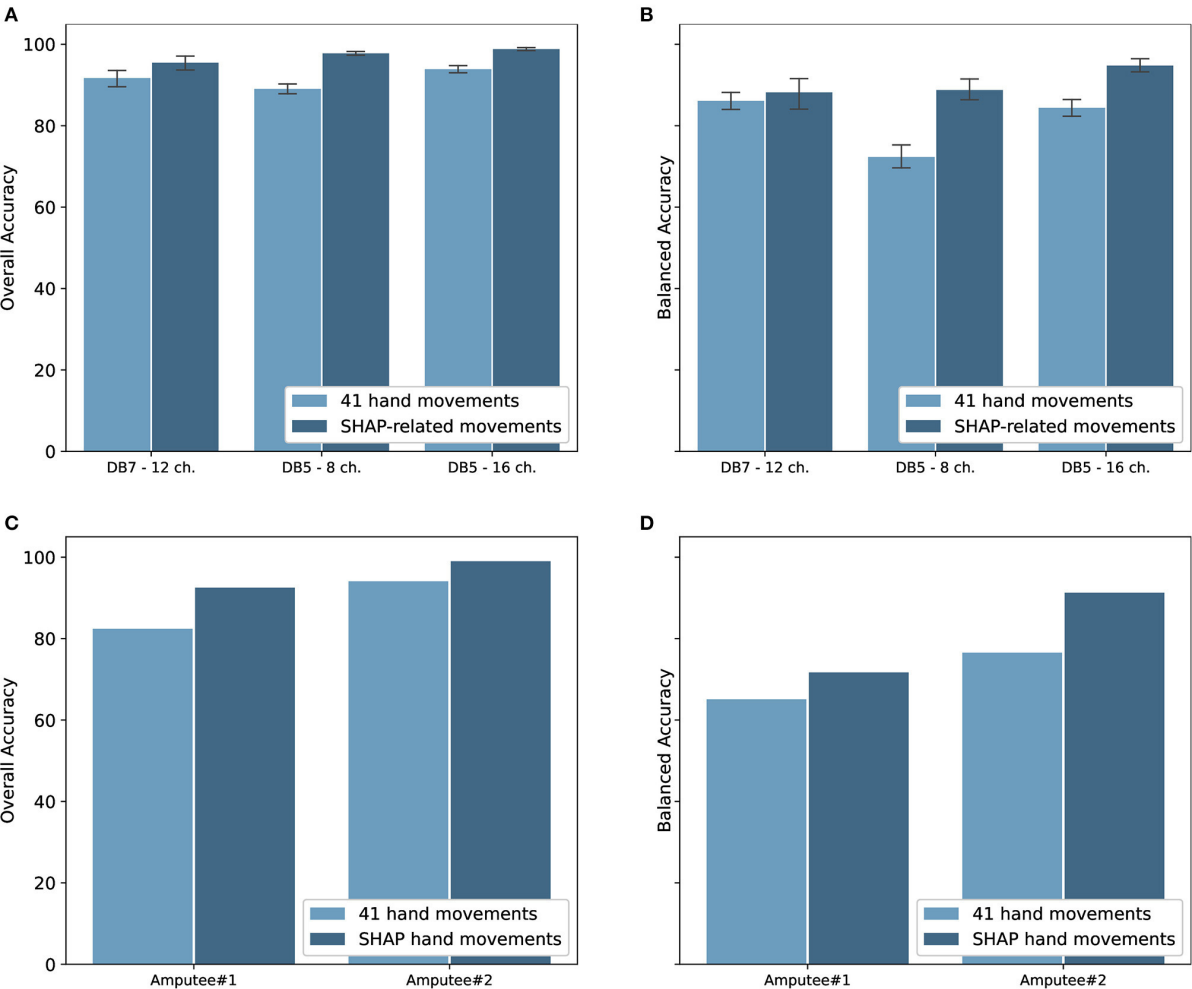
**(A)** Overall accuracy and **(B)** balanced accuracy of the proposed deep neural network classifiers for intact participants; **(C)** overall accuracy and **(D)** balanced accuracy of the classifiers for amputee participants. Note that **(C,D)** do not show standard deviation as there is only one subject shown in each graph.

The classification performance for the intact groups from Ninapro DB5 and DB7 is shown in Figure 6. The experiments were performed on both datasets with window sizes of 100, 200, 400, 800, and 1,000 ms. The stride was set at 100 ms, except for the 100 ms window size in which it was set to 50 ms. For the classification of 41 movements, the proposed model achieved an overall accuracy of 91.69 ± 4.68% and a balanced accuracy of 84.66 ± 4.78% for DB7, the high cost and high sampling rate sensors. An overall accuracy of 93.87 ± 1.49% and a balanced accuracy of 84.00 ± 3.40% were obtained for DB5 with 16 channels, the low cost and low sampling rate sensors. The experiments with only eight channels of the DB5 were also performed. The performance was dropped to an overall accuracy of 89.00 ± 2.05% and balanced accuracy of 71.78 ± 4.67%. Compared to DB5, the balanced accuracy of DB7 is higher, especially for the small window sizes. The complete classification results are available in Supplementary Tables 1–3.

### 3.2. Classification Performance—Amputee Group

Since the goal of the study is to use the movements as classified by the sEMG signals to control the prosthetic hand, our proposed model was validated with the amputee data from the Ninapro DB7. With data from only two amputees available, two instances of our proposed model were trained and validated individually using data from each participant. The experiment results are shown in Figure 6. Data from the amputee participants #1 and #2 achieved notably different results, with overall accuracies of 82.42 and 94.07% and balanced accuracies of 65.10 and 76.55%, respectively.

### 3.3. Classification Performance of SHAP Prehensile Patterns

The Southampton Hand Assessment Procedure (SHAP) is a clinically validated hand functional assessment protocol (Light et al., 2002). It could be used for evaluating the functionality of normal, impaired, or prosthetic hands. The protocol consists of six main prehensile patterns: spherical, tripod, tip, power, lateral, and extension. For our experiments, six movements from the grasping and functional movements group were selected to represent the six SHAP prehensile patterns; power sphere grasp (class 27) for spherical pattern, writing tripod grasp (class 26) for tripod pattern, prismatic pinch grasp (class 31) for tip pattern, large-diameter grasp (class 18) for power pattern, lateral grasp (class 34) for lateral pattern, and extension type grasp (class 36) for extension pattern. The experiment results for the six movements for both intact and amputee groups are shown in Figure 6. There is an evident increase in the performance of the intact group. Our model trained on DB7 and DB5 achieved overall accuracies of 95.78 ± 3.99 and 98.82 ± 0.58% and balanced accuracies of 86.00 ± 8.35 and 94.48 ± 2.55%, respectively. However, a notable improvement is observed for the amputee group. Our model trained on data from amputee #1 and #2 achieved overall accuracies of 92.53 and 99.00% and balanced accuracies of 71.71 and 91.27%, respectively. The complete classification results are available in Supplementary Tables 4, 5.

### 3.4. Classification Performance Analysis

As shown in the previous section, our model achieves higher accuracy on the intact subjects in comparison with the amputee subjects. The results support the hypothesis that the number of subjects is the factor that affects model performance. The more training subjects, the more variance in the movements that the model can generalize with. Since the number of intact subjects is approximately five times more than the amputee subjects, it is possible that gathering data from more amputee subjects may be one logical way to increase accuracy.

For the classification of 41 hand and wrist movements, the best performances were comparable for high sampling rate signal from DB7 and low sampling rate signal from DB5. As expected, the performance of our DB5 dropped significantly when using less channels; the overall accuracy and balanced accuracy of the eight channels setup decreased by 4.87 and 12.22% compared to the 16 channels setup.

**Figure 7:**
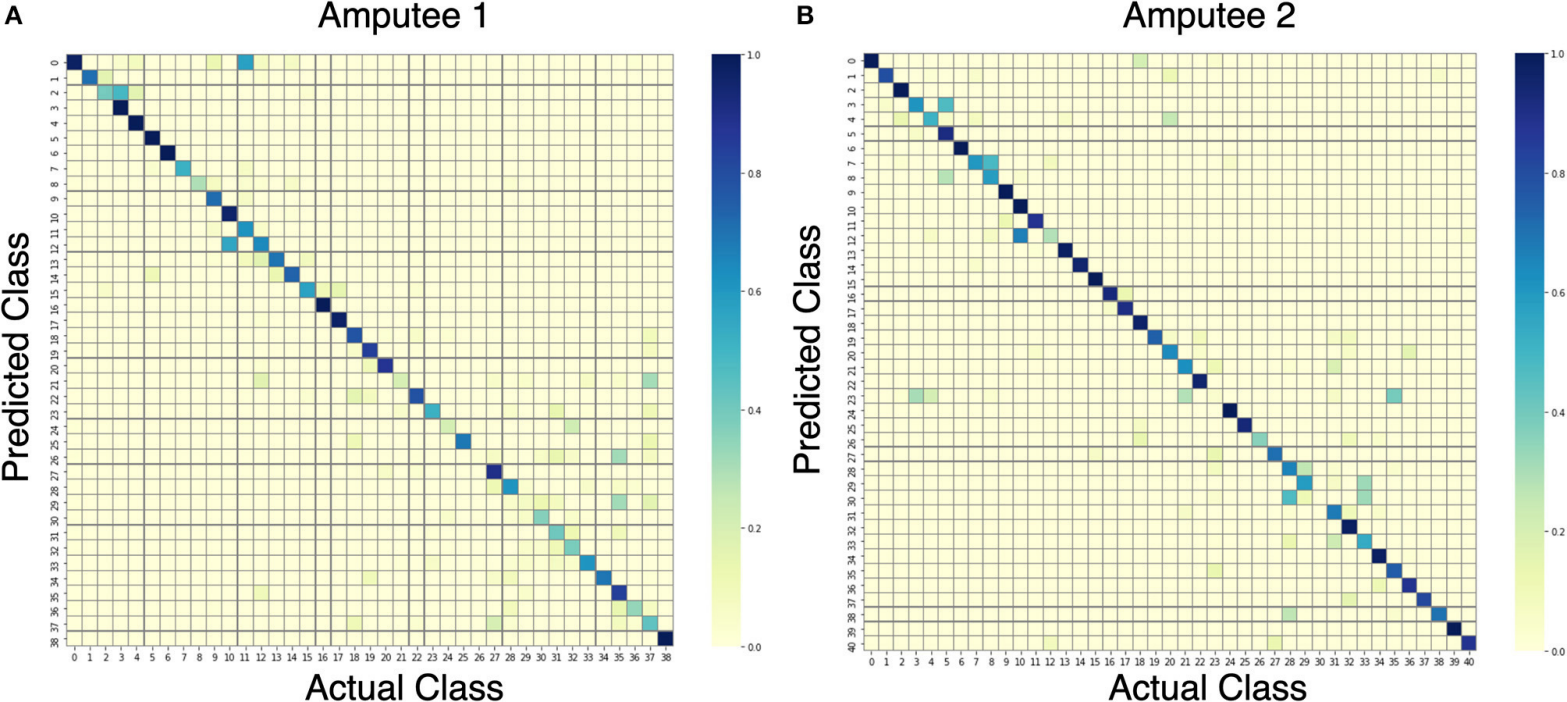
Confusion matrices for the proposed model trained on data from amputees #1 **(A)** and #2 **(B)**.

Upon further analysis of the recall of individual movement classes as shown in Table 2, the results show that our model under-performs with certain hand movements. We consider movements with <80% recall to be difficult to distinguish for practical use. On the other hand, movements with more than 80% recall are considered practical to be classified and thus suitable for production. Out of 10 and 11 movements that are difficult to differentiate for DB7 and DB5, 8 movements are shared among them: 18, 20, 22, 24, 26, 30, 31, and 33. Large diameter grasp (18), fixed hook grasp (20), and medium wrap (22) share many visually similar characteristics. The same could be said for the group of prismatic four fingers grasp (24) and writing tripod grasp (26) movements and the group of tripod grasp (30), prismatic pinch grasp (31), and quadpod grasp (33) movements. Some movements might produce sEMG signals that resemble each other due to the activation of similar groups of muscles and may require further refinement specific to them. The results in Table 2 further demonstrate the difficulty of the classification of 41 movements. For the two amputee participants, there were only 10 movements and 22 movements with more than 80% recall, respectively. A detailed illustration of the classification performance of both amputee participants is presented by the confusion matrix shown in Figure 7.

**Table 2:** Hand movement classes from DB5 and DB7 intact participants, and from DB7 amputees #1 and #2, grouped by recall.

**Intact**		
**Recall**	**DB5—16 channels**	**DB7—12 channels**
≥ 0.8	30 classes: 0, 1, 2, 3, 4,	31 classes: 0, 1, 2, 3, 4,
	5, 6, 7, 8, 9, 10, 13, 14, 15,	5, 6, 7, 8, 9, 10, 11, 12, 13,
	16, 17, 21, 23, 25, 27, 28, 29,	14, 15, 16, 17, 19, 21, 23, 25,
	32, 34, 35, 36, 37, 38, 39, 40	27, 32, 34, 35, 36, 37, 38, 39, 40
<0.6	11 classes: 11, 12, 18, 19,	10 classes:18, 20, 22, 24,
	20, 22, 24, 26, 30, 31, 33	26, 28, 29, 30, 31, 33
**Amputee**		
**Recall**	**Amputee #1**	**Amputee #2**
≥ 0.8	10 classes: 0, 3, 4, 5,	22 classes: 0, 2, 5, 6, 9, 10,
	6, 10, 16, 27, 35, 38	11, 13, 14, 15,16, 17, 18, 22,
		24, 25, 32, 34, 36, 37, 39, 40
0.6 – 0.8	15 classes: 1, 9, 11,	10 classes: 1, 7, 19, 20,
	12, 13, 14, 17, 18, 19,	21, 27, 28, 31, 35, 38
	20, 22, 25, 28, 33, 34	
<0.6	14 classes: 2, 7, 8,	9 classes: 3, 4, 8, 12
	15, 21, 23, 24, 26,	23, 26, 29, 30, 33
	29, 30, 31, 32, 36, 37	

**Figure 8:**
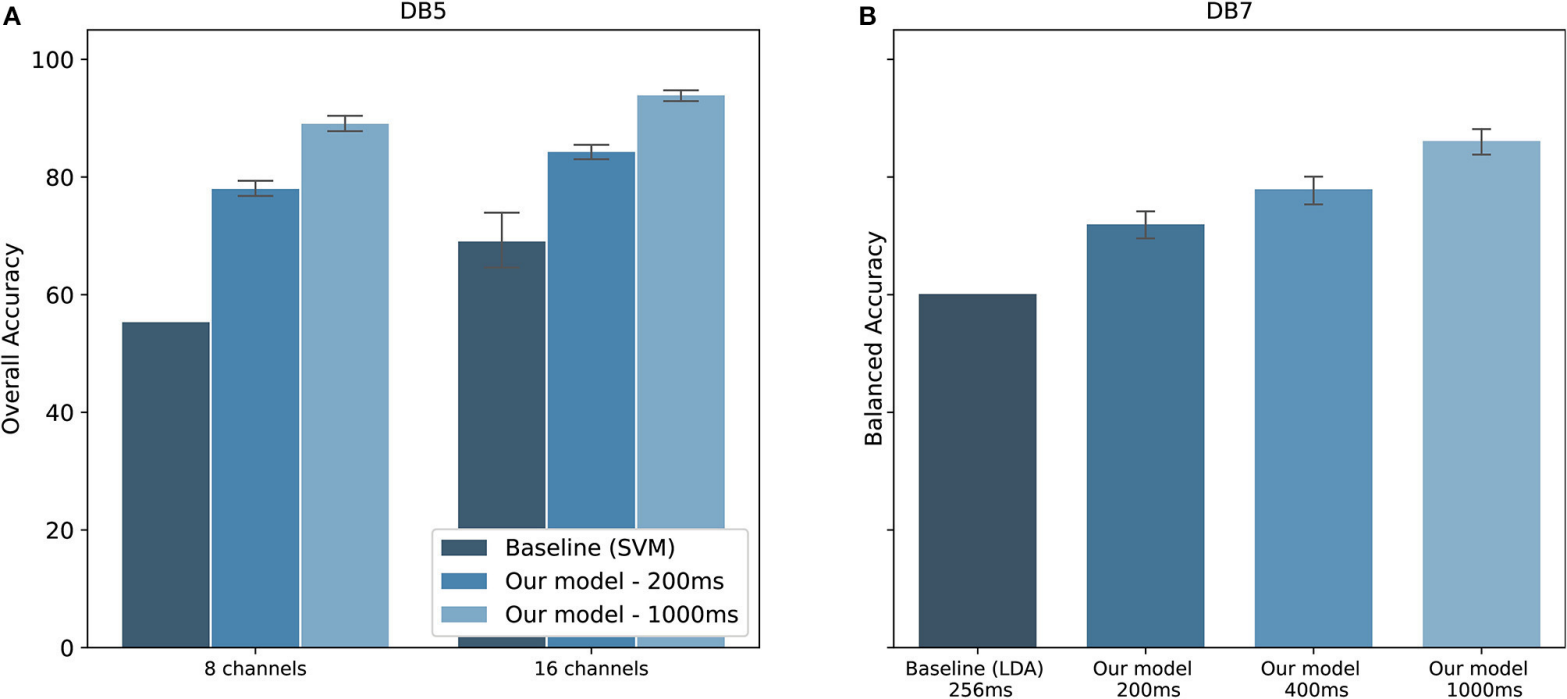
Performance comparison with baseline studies for the classification of 41 hand movements. **(A)** Overall accuracy of our model on DB5 compared to Pizzolato et al. (2017). **(B)** Balanced accuracy on DB7 compared to Krasoulis et al. (2017). The standard deviations for the baseline prior works using eight channels of DB5 and 256 ms of DB7 were not provided.

The performance comparison between our proposed model and the baseline studies are shown in Figure 8. Original studies of the datasets are shown as baselines. For DB5, Pizzolato et al. (2017) reported an overall accuracy of 69.04% for 16 channels and 55.31% for 8 channels. Our model achieved an overall accuracy of 84.25 ± 2.02% for 16 channels and 77.97 ± 2.09% for 8 channels using the same 200 ms window size. The performance was improved by 15.21,and 22.66% for 16 channels and 8 channels, respectively. There is, however, no report on the balanced accuracy. Additionally, a previous study published by our team achieved a balanced accuracy of 77.00% for 1,000 ms window size (Chaiyaroj et al., 2019). With an improvement in the feature extraction and parameter tuning procedures, we achieved a balanced accuracy of 84.00%, observing an increase in performance of 7.00%. For DB7, the study by Krasoulis et al. (2017) reached a balanced accuracy of 60.10% by using sEMG data and 256 ms window size. Our model reported a balanced accuracy of 70.67 ± 5.47, 75.45 ± 5.31, and 84.66 ± 4.78% for the window size of 200, 400, and 1,000 ms, respectively. One important point from the original study is that the balanced accuracy could reach 82.70% with the inclusion of additional inertial measurement sensors.

**Figure 9:**
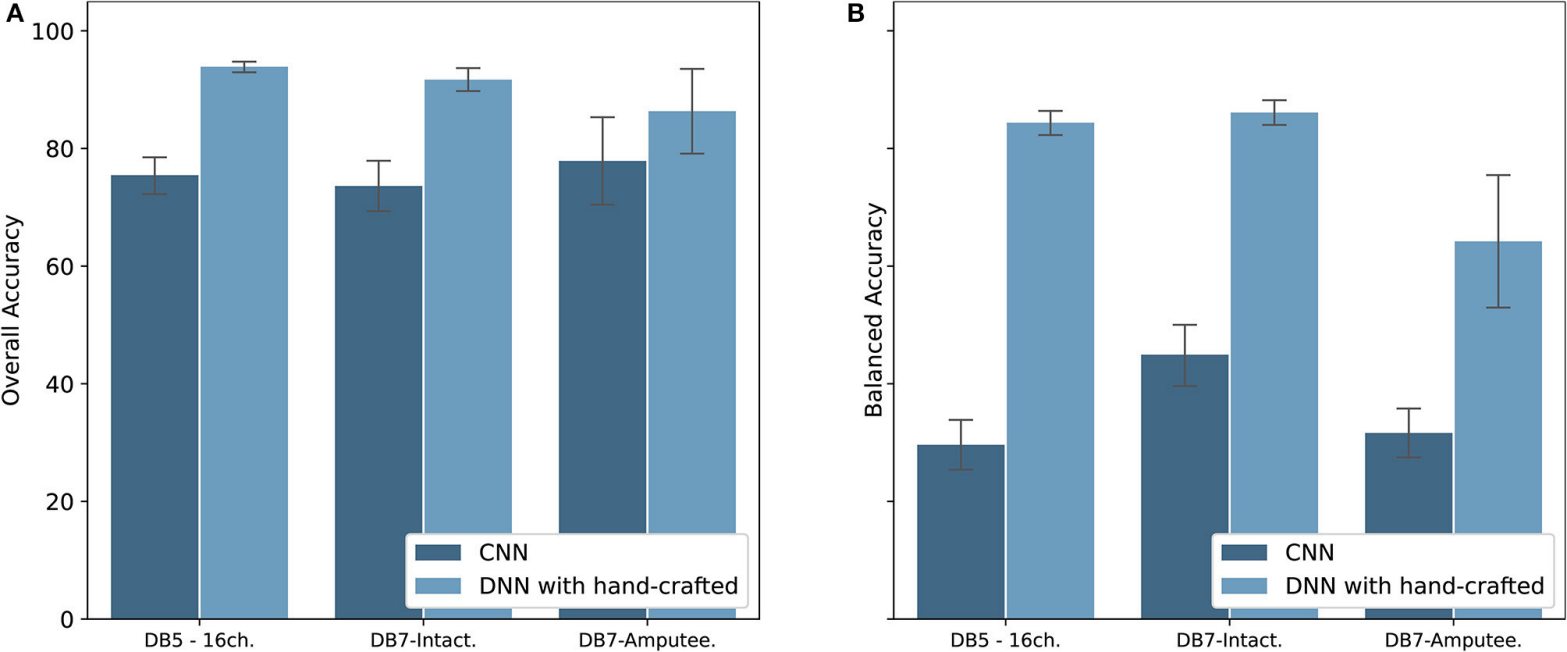
Overall accuracy **(A)** and Balanced accuracy **(B)** comparison between CNN and DNN with hand-crafted features for DB5 with 16 channels, intact group of DB7, and amputee group of DB7.

With recent research highlighting the performance of CNN for the classification of hand movements (Park and Lee, 2016; Ameri et al., 2018; Tsinganos et al., 2018), we implemented a CNN model to be compared with our proposed model. The results are shown in Figure 9. Our method of extracting hand-crafted features and feed them to DNN outperforms in both overall and balanced accuracy. The proposed model achieved an overall accuracy of 93.87 ± 1.49, 91.69 ± 4.68, and 86.33 ± 7.20 and balanced accuracy of 84.00 ± 3.40, 84.66 ± 4.78, and 64.22 ± 11.27 for DB5 with 16 channels, intact group of DB7, and amputee group of DB7, respectively. Our CNN achieved an overall accuracy of 75.45 ± 3.62, 73.62 ± 4.81, and 77.88 ± 7.43 and balanced accuracy of 29.62 ± 4.75, 44.95 ± 6.01, and 31.63 ± 4.16 for DB5 with 16 channels, intact group of DB7, and amputee group of DB7, respectively. Considering prior works use <10 movements, we suspect that our dataset contains too few samples for CNN to learn to classify all 41 movement classes effectively.

### 3.5. SHAP Prehensile Patterns Performance Analysis

For the classification of six movements based on SHAP prehensile patterns, the low sampling rate sensors setup with data from DB5 outperformed the DB7 setup even when using only eight channels. A balanced accuracy of the DB5 setups with 16 channels and eight channels was 8.48 and 2.61% higher than the DB7 setup, respectively. According to these experiment results, for a certain set of movements, a 200 Hz sampling rate sensor with eight sEMG input channels could be enough to achieve an accurate result. As for the amputee participants, the setup for amputee #2 achieved an over 14.72% boost, resulting in a balanced accuracy of 91.27%. Therefore, finding a balance between the number of movements, the number of sensors, and the sensor sampling rate is the key to optimizing the performance of the prosthesis.

### 3.6. Usage Simulation Analysis

**Figure 10:**
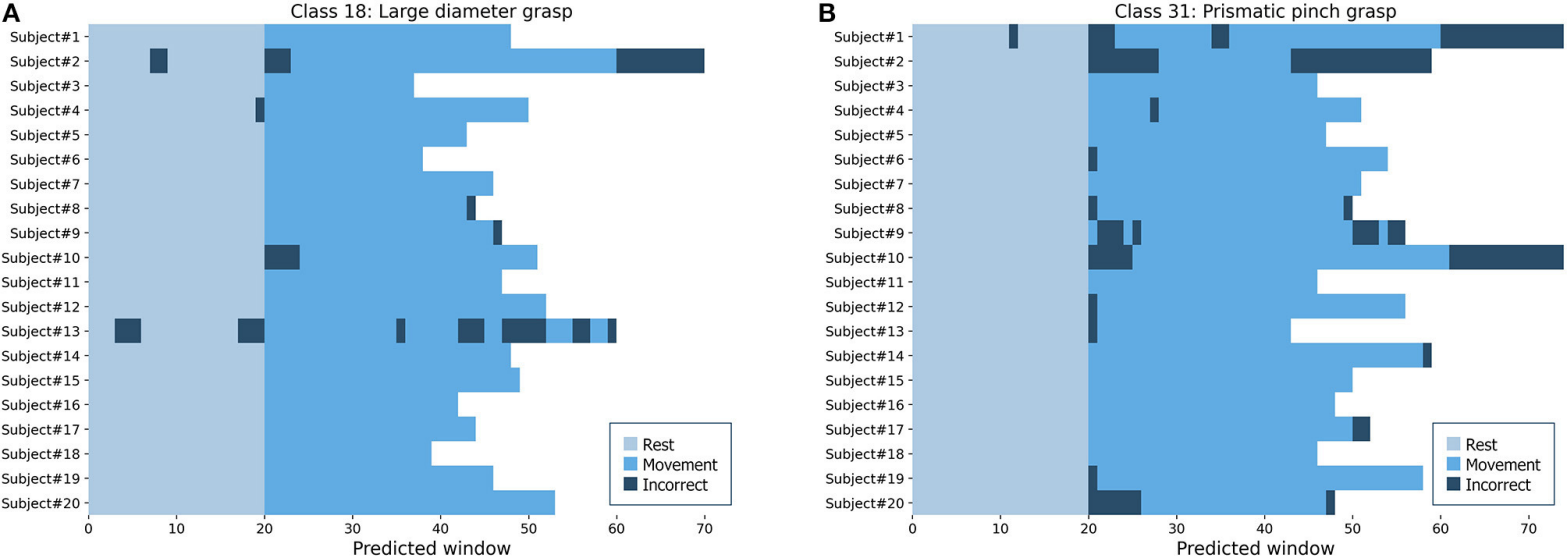
Predicted sequences of full repetitions for every intact participant from DB7 **(A)** the large-diameter grasp (class 18) **(B)** the prismatic pinch grab (class 31).

To simulate the performance in a real setting, we used the proposed model to predict the hand and wrist movements as full sequences from the complete lengths of sEMG signals in each repetition from intact subjects. Figure 10 shows two examples of predicted sequences of the large-diameter grasp (class 18) as a basic grasping functional movement and the prismatic pinch grab (class 31) as one of the SHAP hand movements. The figure illustrates the full movement period, as well as 20 windows of the preceding rest period, of each intact participant's fifth repetitions from DB7. Most of the movements were predicted in long and continuous spans, from which we can infer that it would lead to successful and smooth movements. Wrong predictions at the beginning or the end of the periods could be explained as transitional moments between rest and movement, which our model was not trained to handle directly. Such errors in the middle, however, indicate moments where a prosthetic hand may abruptly switch to an unintended movement. Further development for the implementation of the proposed model into the prosthetic hand system could include the post-processing step to minimize the effect of prediction errors to ensure user-friendliness and safety.

## 4. Discussion

### 4.1. Window Size Comparison

Response time is one important factor for a user's acceptance of the prosthetic hand. Longer response time might lead to unsatisfactory performance. However, frequent instant responses with inaccurate movements could lead to frustration and even rejection of the prosthesis. Therefore, achieving the balance between response time and reliability or accuracy of the classification is crucial for the development of the prosthetic hand. For our approach, the key parameters that contribute to a model's computation complexity are the window size and stride. Large window size requires more computation power and memory and increases the response time. Stride, on the other hand, reflects the frequency at which the model makes decisions; therefore, a smaller stride would increase the processor's activity rate, and thus computation cost as well as power consumption. For real-time decisions, majority voting is usually considered to be an effective strategy that can increase overall reliability (Geng et al., 2016; Menon et al., 2017). Therefore, smaller stride can also mean faster response time in some cases. To meet the hardware constraints, tuning these two parameters is a necessary step for the development of the prosthesis. Oskoei and Hu (2007) reported a detailed experiment on the relationship between window size and classification accuracy, including the window size and stride.

For our experiment, the effect of the window size is shown in the detailed performance in Tables 3, 4. The larger the window size is, the higher the performance gain is achieved by our model. For the intact group from DB7, the proposed model achieved an overall accuracy between 82.83 and 91.69% with a balanced accuracy between 66.73 and 84.66% across all window sizes. DB5 with 16 channels achieved an overall accuracy between 81.37 and 93.87% with a balanced accuracy between 55.10 and 84.00% while DB5 with eight channels achieved an overall accuracy between 74.00 and 89.00% with a balanced accuracy between 36.14 and 71.78%. Based on these experiments, the high sampling rate sensors setup performed remarkably better than the low sampling rate sensors setup for small window sizes. The performances for the larger window sizes are comparable. We suspect that the sampling rate of 200 Hz with small window sizes might not have enough information to distinguish the subtle differences between 41 movements.

**Table 3:** Accuracy and macro-averaged metrics of the proposed deep neural network classifiers for 41 hand movements.

	**Accuracy**	**Macro-precision**	**Macro-recall**	**Macro-F1**
**DB5—8 channels : intact participants**				
100 ms	74.00 ± 2.10	42.79 ± 4.40	36.14 ± 4.80	38.68 ± 4.70
200 ms	77.97 ± 2.09	51.58 ± 4.73	46.13 ± 4.56	47.95 ± 4.69
400 ms	80.88 ± 1.99	56.44 ± 4.12	52.48 ± 4.55	53.85 ± 4.49
800 ms	87.04 ± 1.83	68.88 ± 4.08	67.56 ± 4.01	68.00 ± 4.06
1,000 ms	89.00 ± 2.05	73.32 ± 4.11	71.78 ± 4.67	72.35 ± 4.54
**DB5—16 channels : intact participants**					
100 ms	81.37 ± 2.17	59.43 ± 5.13	55.10 ± 5.00	56.90 ± 5.03
200 ms	84.25 ± 2.02	65.21 ± 4.48	62.07 ± 4.56	63.38 ± 4.57
400 ms	87.21 ± 1.86	71.69 ± 3.68	68.54 ± 4.43	69.70 ± 4.31
800 ms	90.72 ± 1.62	77.45 ± 3.34	76.88 ± 3.84	76.88 ± 3.75
1,000 ms	93.87 ± 1.49	85.57 ± 2.46	84.00 ± 3.40	84.67 ± 3.20
**DB7—12 channels : intact participants**					
100 ms	82.83 ± 4.90	73.96 ± 3.21	66.73 ± 4.89	69.78 ± 4.05
200 ms	85.08 ± 4.83	78.83 ± 2.88	70.67 ± 5.47	74.18 ± 4.38
400 ms	87.74 ± 4.94	81.81 ± 3.29	76.56 ± 5.31	78.90 ± 4.47
800 ms	90.61 ± 4.73	85.48 ± 3.55	82.30 ± 5.10	83.77 ± 4.50
1,000 ms	91.69 ± 4.68	87.03 ± 4.06	84.66 ± 4.78	85.74 ± 4.55
**DB7—12 channels : amputee #1**					
100 ms	74.64	55.27	48.20	50.18
200 ms	78.23	63.91	53.79	56.05
400 ms	79.16	64.55	57.56	58.44
800 ms	81.54	69.97	62.02	63.08
1000 ms	82.42	72.84	65.10	65.66
**DB7—12 channels : amputee #2**					
100 ms	87.49	63.62	59.23	60.26
200 ms	89.68	69.20	65.33	66.23
400 ms	89.01	64.25	62.39	61.66
800 ms	93.41	76.39	74.87	74.35
1,000 ms	94.07	75.78	76.55	74.90

**Table 4:** Accuracy and macro-averaged metrics of the proposed deep neural network classifiers for SHAP prehensile patterns.

	**Accuracy**	**Macro-precision**	**Macro-recall**	**Macro-F1**
**DB5—8 channels : intact participants**				
100 ms	92.49 ± 1.26	69.16 ± 4.13	61.21 ± 6.18	64.71 ± 5.00
200 ms	93.71 ± 1.11	72.81 ± 4.26	68.59 ± 5.72	70.52 ± 4.79
400 ms	95.53 ± 1.24	80.56 ± 5.58	77.42 ± 6.24	78.76 ± 5.91
800 ms	97.29 ± 0.86	87.17 ± 2.90	86.27 ± 4.69	86.61 ± 3.94
1,000 ms	97.78 ± 0.75	89.14 ± 3.58	88.61 ± 3.95	88.93 ± 3.48
**DB5—16 channels : intact participants**				
100 ms	94.66 ± 1.05	79.40 ± 3.23	74.14 ± 5.50	76.63 ± 4.22
200 ms	95.86 ± 0.89	82.74 ± 2.78	80.96 ± 4.37	81.79 ± 3.55
400 ms	97.28 ± 0.81	89.49 ± 2.34	87.10 ± 4.01	88.19 ± 3.05
800 ms	98.38 ± 0.84	93.17 ± 3.59	92.10 ± 3.95	92.60 ± 3.80
1,000 ms	98.82 ± 0.58	94.93 ± 1.91	94.48 ± 2.55	94.67 ± 2.18
**DB7—12 channels : intact participants**					
100 ms	92.69 ± 4.19	86.42 ± 3.96	74.08 ± 9.32	79.40 ± 7.32
200 ms	93.34 ± 4.41	88.01 ± 4.02	76.81 ± 10.05	81.66 ± 7.86
400 ms	94.22 ± 4.03	88.90 ± 4.00	79.92 ± 8.91	83.77 ± 7.28
800 ms	95.46 ± 4.16	90.30 ± 4.38	85.18 ± 8.90	87.59 ± 7.03
1,000 ms	95.78 ± 3.99	90.94 ± 3.90	86.00 ± 8.35	88.31 ± 6.79
**DB7—12 channels : amputee #1**					
100 ms	88.29	68.02	56.73	59.82
200 ms	88.88	69.94	56.66	58.09
400 ms	90.20	76.42	63.87	65.71
800 ms	91.14	77.48	67.39	69.22
1,000 ms	92.53	83.22	71.71	74.97
**DB7—12 channels : amputee #2**					
100 ms	97.13	87.43	80.08	81.83
200 ms	97.55	87.11	83.99	83.38
400 ms	98.04	91.95	84.44	85.47
800 ms	98.66	94.93	88.92	89.92
1,000 ms	99.00	96.24	91.27	93.11

For the six movements based on SHAP prehensile patterns, surprisingly, the intact group from DB5 with 16 channels performed considerably better than DB7 on every window size. DB7 achieved a balanced accuracy between 74.08 and 86.00% while DB5 with 16 channels achieved a balanced accuracy between 74.14 and 94.48%, approximately 7% better for the window sizes of 400 ms or more. For the classification of several movements, a higher number of input channels might be more important than a higher sampling rate. Another interesting point is that the performances of the setups on DB5 with eight channels and DB7 are comparable for the window size of 400 ms or more. Therefore, based on these experiments, the sampling rate of 200 Hz with eight channels of the sEMG input could be enough for the classification of six movements.

The experiment results for the amputee participants were similar to the intact group. Within the extent of the dataset, the six movements setup for amputee #2 achieved outstanding results on all window sizes, with an overall accuracy between 97.13 and 99.00% and balanced accuracy between 80.08 and 91.27%. However, the conclusive decision for the window size ultimately depends on the hardware, tasks, and even the user.

### 4.2. Running Time

The classification model and window size affect computational complexity and running time. The slow response of a large model can cause an uncomfortable usage experience. On the other hand, a smaller model yields less accuracy and might require multiple takes to be able to identify the correct movement. Therefore, due to hardware limitations, there is an important trade-off between accuracy and computation speed.

For the setup of computational resources, our model was implemented, trained, tuned, and evaluated on the Google Colaboratory platform with Intel(R) Xeon(R) CPU @ 2.30 GHz CPU, 12 GB of RAM, and Tesla P100-PCIE-16GB GPU. The model was run on Python 3.6.8, Keras 2.2.5, and Tensorflow 1.14.0. To illustrate the effect of the models and window sizes, model decision time per sample is shown in Table 5. The larger window size means more computation resulting in longer decision time. To implement the model into a prosthetic hand, the model needs to run on the embedded processor which will be substantially less powerful. However, current technology is heading in a direction that may bridge this trade-off; edge computing hardware equipped with GPU, such as NVIDIA's Jetson Nano, is already available commercially, and may soon become one of the solutions to bringing deep neural networks into the field of the prosthesis.

**Table 5:** Average prediction time per sample of the proposed deep neural network model.

	**Prediction time/sample (picosecond)**
100 ms	35.89
200 ms	53.43
400 ms	76.75
800 ms	89.85
1,000 ms	98.86

## 5. Conclusion

This study presents an application of a deep neural network model for classifying 41 hand movements based on surface electromyogram. The public datasets Ninapro DB5 and DB7 were used as low sampling rate data and high sampling rate data for our experiment. The acquisition setup for DB5 was based on two Thalmic Myo armbands with 16 channels of input and 200 Hz sampling rate, while DB7 was recorded by Delsys Trigno electrodes with 12 channels of input and 2 kHz sampling rate. Following the Southhampton Hand Assessment Procedure (SHAP), we also performed experiments for the classification of the six movements based on six prehensile patterns for hand functionality evaluation. Compared to other studies' classification results, our proposed model outperformed the best results of the previous studies from Pizzolato et al. (2017) and Krasoulis et al. (2017). This is a promising result, though some confirmation from a larger experiment with more data samples would certainly be beneficial. Experimentation on the window size shows that the larger the window size is, the higher the performance gain the proposed model achieves, which is expected. Lastly, we measured the running time of our proposed model to compare the feasibility of using different window sizes. We believe that given sufficient data, our proposal could be a feasible approach for controlling advanced prosthetic hands.

## Data Availability Statement

The original datasets are from Ninapro DB5 and DB7. The preprocessed datasets generated for this study are available on request to the corresponding author.

## Ethics Statement

The studies involving human participants were reviewed and approved by the Local Ethics Committees of the School of Informatics, University of Edinburgh and School of Electrical and Electronic Engineering, Newcastle University for Ninapro DB7 as stated by Krasoulis et al. (2017). For Ninapro DB5, all experiments were approved by the Ethics Commission of the Canton of Valais, Switzerland as stated by Pizzolato et al. (2017). The patients/participants provided their written informed consent to participate in this study.

## Author Contributions

PS and AC performed the experiments, data analysis, and manuscript preparation. CB, SD, RP, and CT were involved in the discussions regarding the experiments. DS was PI, designed the experiments, and was involved in all facets of the study and manuscript preparation. All authors approved the final manuscript.

## Conflict of Interest

The authors declare that the research was conducted in the absence of any commercial or financial relationships that could be construed as a potential conflict of interest.
