# Supplementary material for: Classification of 41 Hand and Wrist Movements via Surface Electromyogram Using Deep Neural Network
Source: Front Bioeng Biotechnol. 2021 Jun 9;9:548357. doi: 10.3389/fbioe.2021.548357 (PMC8220079; doi:10.3389/fbioe.2021.548357)
Supplement: Supplementary file 1 [file Data_Sheet_1.PDF]

Supplementary Material

1 SUPPLEMENTARY DATA

Table S1. DB5 16-Channel (1000 ms)

Class	Precision	Recall	F1	Class	Precision	Recall	F1
0	98.66	99.60	99.13	21	92.89	89.78	91.31
1	89.17	90.81	89.98	22	73.13	78.19	75.58
2	94.00	87.98	90.89	23	87.52	86.75	87.13
3	93.65	96.63	95.12	24	91.08	75.90	82.80
4	84.80	87.43	86.10	25	84.20	89.32	86.68
5	86.95	82.16	84.48	26	87.09	72.63	79.20
6	90.38	92.90	91.62	27	81.65	86.08	83.80
7	93.74	87.13	90.31	28	78.84	84.82	81.72
8	84.43	81.65	83.02	29	78.69	85.47	81.94
9	89.55	86.59	88.04	30	84.95	71.95	77.91
10	77.74	81.03	79.35	31	81.35	68.23	74.21
11	80.76	76.24	78.43	32	76.70	81.80	79.17
12	72.35	79.55	75.78	33	79.08	71.36	75.02
13	86.26	84.71	85.48	34	91.28	87.87	89.54
14	87.91	93.67	90.70	35	87.68	83.63	85.61
15	89.03	84.30	86.60	36	87.62	88.46	88.04
16	83.97	80.40	82.14	37	95.04	90.86	92.91
17	85.32	88.64	86.95	38	84.51	82.74	83.62
18	73.24	73.54	73.39	39	92.28	92.78	92.53
19	73.67	75.19	74.42	40	96.02	93.20	94.59
20	81.00	72.04	76.26				
				**Macro-Average**	85.57	84.00	84.67
				**Micro-Average**	93.84	93.87	93.81

Table S2. DB5 8-Channel (1000 ms)

Class	Precision	Recall	F1	Class	Precision	Recall	F1
0	98.19	99.09	98.64	21	81.35	59.14	68.49
1	80.12	85.15	82.55	22	60.48	66.49	63.34
2	71.38	79.26	75.11	23	79.28	71.58	75.23
3	86.43	87.08	86.75	24	67.08	76.10	71.31
4	79.96	81.04	80.50	25	77.51	72.38	74.86
5	83.49	76.87	80.05	26	72.68	55.84	63.16
6	85.14	82.58	83.84	27	67.03	65.82	66.42
7	68.85	76.01	72.25	28	76.24	56.83	65.12
8	71.27	65.98	68.52	29	60.75	54.90	57.68
9	73.37	70.56	71.94	30	55.59	60.66	58.01
10	71.46	66.85	69.08	31	55.59	64.19	59.58
11	63.11	59.78	61.40	32	58.62	58.88	58.75
12	59.01	66.98	62.74	33	60.21	57.12	58.62
13	86.67	77.16	81.64	34	85.56	74.65	79.73
14	82.52	81.44	81.98	35	79.17	74.11	76.56
15	77.72	77.54	77.63	36	77.48	78.85	78.16
16	74.60	71.43	72.98	37	83.07	78.85	80.90
17	77.65	77.12	77.38	38	67.29	75.32	71.08
18	64.02	68.28	66.08	39	84.83	83.79	84.30
19	58.04	68.66	62.91	40	84.44	84.67	84.55
20	58.74	54.17	56.36				
				**Macro-Average**	73.32	71.78	72.35
				**Micro-Average**	89.01	89.00	88.93

Table S3. DB7 Intact (1000 ms)

Class	Precision	Recall	F1	Class	Precision	Recall	F1
0	95.68	97.99	96.82	21	88.18	86.44	87.30
1	89.39	92.09	90.72	22	80.28	76.94	78.58
2	89.34	91.18	90.25	23	89.05	89.12	89.09
3	92.65	94.09	93.37	24	79.36	70.85	74.87
4	93.41	88.90	91.10	25	86.48	85.05	85.76
5	90.10	86.50	88.27	26	79.17	65.53	71.71
6	90.91	94.92	92.87	27	89.23	84.46	86.78
7	93.30	81.20	86.83	28	82.80	70.98	76.43
8	86.03	84.15	85.08	29	72.12	63.71	67.66
9	87.52	84.88	86.18	30	70.32	74.87	72.52
10	86.35	88.81	87.56	31	90.02	79.06	84.18
11	86.00	80.94	83.39	32	81.04	80.66	80.85
12	85.70	86.78	86.24	33	74.04	76.77	75.38
13	90.60	91.74	91.16	34	83.38	83.85	83.61
14	93.91	92.66	93.28	35	94.16	90.72	92.41
15	93.77	89.31	91.48	36	94.60	90.16	92.33
16	84.24	85.34	84.79	37	93.77	93.46	93.62
17	90.34	85.91	88.07	38	89.72	92.79	91.23
18	86.54	76.61	81.27	39	95.43	92.76	94.08
19	71.44	80.69	75.78	40	94.89	95.10	94.99
20	82.78	73.06	77.62				
				**Macro-Average**	87.03	84.66	85.74
				**Micro-Average**	91.57	91.69	91.58

Table S4. DB7 Amputee #1 (1000 ms)

Class	Precision	Recall	F1	Class	Precision	Recall	F1
0	88.80	98.03	93.19	21	77.78	20.29	32.18
1	100.00	71.05	83.08	22	80.60	78.26	79.41
2	65.52	39.58	49.35	23	87.10	51.92	65.06
3	65.43	100.00	79.10	24	68.42	20.47	31.52
4	80.43	100.00	89.16	25	97.37	68.52	80.43
5	87.88	100.00	93.55	26	40.00	2.02	3.85
6	100.00	100.00	100.00	27	65.06	90.00	75.52
7	100.00	51.90	68.33	28	59.42	61.19	60.29
8	95.83	29.49	45.10	29	20.00	6.82	10.17
9	71.60	71.60	71.60	30	60.78	36.47	45.59
10	63.41	96.30	76.47	31	43.21	40.70	41.92
11	42.22	61.96	50.22	32	44.30	38.46	41.18
12	50.00	64.29	56.25	33	78.26	61.02	68.57
13	82.76	69.57	75.59	34	100.00	69.39	81.93
14	84.13	73.61	78.52	35	44.79	84.31	58.50
15	80.00	57.14	66.67	36	78.57	33.67	47.14
16	90.62	100.00	95.08	37	29.63	43.64	35.29
17	86.84	97.06	91.67	38	98.44	100.00	99.21
18	64.79	77.97	70.77				
19	75.44	84.31	79.63				
20	91.49	87.76	89.58				
				**Macro-Average**	72.84	65.10	65.66
				**Micro-Average**	81.63	82.42	80.30

Table S5. DB7 Amputee #2 (1000 ms)

Class	Precision	Recall	F1	Class	Precision	Recall	F1
0	99.50	99.84	99.67	21	64.71	62.86	63.77
1	94.59	79.55	86.42	22	88.00	95.65	91.67
2	84.44	100.00	91.57	23	6.45	3.85	4.82
3	61.22	61.22	61.22	24	95.12	100.00	97.50
4	62.50	51.02	56.18	25	100.00	93.18	96.47
5	53.23	91.67	67.35	26	100.00	36.00	52.94
6	100.00	100.00	100.00	27	84.62	70.97	77.19
7	78.95	60.00	68.18	28	41.67	65.79	51.02
8	50.00	58.62	53.97	29	61.54	59.26	60.38
9	89.36	100.00	94.38	30	0.00	0.00	0.00
10	58.70	100.00	73.97	31	51.22	67.74	58.33
11	92.68	88.37	90.48	32	67.53	98.11	80.00
12	62.50	28.57	39.22	33	45.45	53.57	49.18
13	94.55	98.11	96.30	34	83.02	97.78	89.80
14	100.00	95.56	97.73	35	61.76	75.00	67.74
15	94.12	100.00	96.97	36	82.98	88.64	85.71
16	94.34	92.59	93.46	37	100.00	81.40	89.74
17	87.88	90.62	89.23	38	92.86	70.27	80.00
18	70.00	96.55	81.16	39	100.00	100.00	100.00
19	94.59	74.47	83.33	40	96.23	87.93	91.89
20	60.53	63.89	62.16				
				**Macro-Average**	75.78	76.55	74.90
				**Micro-Average**	93.96	94.07	93.69
